# Depression, anxiety, and quality of life as predictors of rehospitalization in patients with chronic heart failure

**DOI:** 10.1186/s12872-023-03500-8

**Published:** 2023-10-27

**Authors:** Jovan Veskovic, Mina Cvetkovic, Elvis Tahirovic, Marija Zdravkovic, Svetlana Apostolovic, Dragana Kosevic, Goran Loncar, Danilo Obradovic, Dragan Matic, Aleksandra Ignjatovic, Tatjana Cvetkovic, Maximilian G. Posch, Sara Radenovic, Arsen D. Ristić, Danilo Dokic, Nenad Milošević, Natasa Panic, Hans-Dirk Düngen

**Affiliations:** 1 Department of Internal Medicine, Cardiology, CVK, Charité University Medicine Berlin, 13353 Berlin, Germany; 2 German Centre for Cardiovascular Research (DZHK), Partner Site Berlin, 13353 Berlin, Germany; 3 Department of Cardiology, Faculty of Medicine, University Clinical Hospital Center Bezanijska Kosa, University of Belgrade, Belgrade, 11000 Serbia; 4 Department for Cardiovascular Diseases, Clinical Centre Niš, University of Niš, Niš, 18000 Serbia; 5 Institute for Cardiovascular Diseases Dedinje, Department of Cardiology, Belgrade, 11000 Serbia; 6 Faculty of Medicine, Department of Cardiology, University of Belgrade, University Clinical Center of Serbia, Belgrade, 11000 Serbia; 7 Heart Center of Leipzig, University of Leipzig, 04289 Leipzig, Germany; 8 Department of Cardiology, University Clinical Centre of Serbia, Belgrade, 11000 Serbia; 9 Faculty of Medicine, University of Niš, Niš, 18000 Serbia; 10 Scirent Clinical Research and Science, 13353 Berlin, Germany

**Keywords:** Depression, Anxiety, Quality of life, Rehospitalization, Prediction, Heart failure

## Abstract

**Background:**

Chronic heart failure (CHF) is a severe condition, often co-occurring with depression and anxiety, that strongly affects the quality of life (QoL) in some patients. Conversely, depressive and anxiety symptoms are associated with a 2–3 fold increase in mortality risk and were shown to act independently of typical risk factors in CHF progression. The aim of this study was to examine the impact of depression, anxiety, and QoL on the occurrence of rehospitalization within one year after discharge in CHF patients.

**Methods:**

148 CHF patients were enrolled in a 10-center, prospective, observational study. All patients completed two questionnaires, the Hospital Anxiety and Depression Scale (HADS) and the Questionnaire Short Form Health Survey 36 (SF-36) at discharge timepoint.

**Results:**

It was found that demographic and clinical characteristics are not associated with rehospitalization. Still, the levels of depression correlated with gender (p ≤ 0.027) and marital status (p ≤ 0.001), while the anxiety values ​​were dependent on the occurrence of chronic obstructive pulmonary disease (COPD). However, levels of depression (HADS-Depression) and anxiety (HADS-Anxiety) did not correlate with the risk of rehospitalization. Univariate logistic regression analysis results showed that rehospitalized patients had significantly lower levels of Bodily pain (BP, p = 0.014), Vitality (VT, p = 0.005), Social Functioning (SF, p = 0.007), and General Health (GH, p = 0.002). In the multivariate model, poor GH (OR 0.966, p = 0.005) remained a significant risk factor for rehospitalization, and poor General Health is singled out as the most reliable prognostic parameter for rehospitalization (AUC = 0.665, P = 0.002).

**Conclusion:**

Taken together, our results suggest that QoL assessment complements clinical prognostic markers to identify CHF patients at high risk for adverse events. **Clinical Trial Registration**: The study is registered under http://clinicaltrials.gov (NCT01501981, first posted on 30/12/2011), sponsored by Charité – Universitätsmedizin Berlin.

## Background

Chronic heart failure (CHF) is a severe, progressive, and incurable condition characterized by dyspnea, edema, fluid retention, pulmonary congestion, and fatigue due to a structural and/or functional abnormality of the heart resulting in inadequate cardiac output. The term CHF describes patients with an established diagnosis of heart failure (HF) and/or those who experience more gradual onset of symptoms compared to acute HF [1]. In addition, CHF may be complicated by depression and anxiety, further affecting the quality of patients’ lives. Psycho-social factors may also contribute to the poor prognosis of heart failure (HF) patients [2]. Depression is up to 5 times more common in CHF patients relevant to the general population, with a prevalence of 21,5% [3], and it doubles the mortality risk independently of biological (classical) risk factors (comorbidities including coronary artery disease, diabetes, hypertension, obesity, age, family history, social isolation, psychological stress etc.) [4–6]. The physiological mechanisms underlying depression and heart failure have shared elements. Indeed, factors that worsen cardiac function in patients with heart failure are also common in patients with depressive disorder: a hyperactive hypothalamic–pituitary–adrenal axis, decreased heart rate variability, higher levels of inflammatory markers, high-risk behavior (smoking, more sedentary lifestyle), lower adherence with medication and dietary guidelines, abnormalities in platelet function, and weaker social support [5, 7–10].

Anxiety symptoms are also highly prevalent in CHF. About 13% of CHF patients meet the diagnostic requirements for generalized anxiety disorder, and almost 30% of patients have confirmed anxiety (based on different anxiety questionnaires) [11, 12]. Additionally, the prevalence of anxiety symptoms in CHF spans between 38 and 70% in different study populations [12, 13]. Many guidelines recognize the relevance of psychological problems associated with CHF and recommend regular monitoring for symptoms of depression and anxiety in patients [14]. Past research has reported that the presence of anxiety symptoms is an independent predictor for: (1) deteriorating social functioning status, (2) health-associated quality of life, and (3) increased rehospitalizations in as many as one-third of all patients with HF [15]. A study by Mulle and colleagues suggested that the combination of depression and anxiety enhances the risk for incident CHF and found that these two pathological conditions share common genetic factors [16]. Further investigations revealed a genetic overlap of depression and anxiety phenotypes, particularly in women [16, 17].

Quality of life (QoL) is an important parameter in evaluating therapeutic strategies for multiple diseases. It is known that CHF is associated with severely impaired QoL [18]. Furthermore, QoL progressively decreases in patients as NYHA functional class worsen [19]. Patients with heart failure have various physical and emotional symptoms that limit them in daily physical and social activities and result in poor QoL [17–22].

Mental health diseases are associated with frequent hospitalizations and an increased risk of all - cause mortality. Despite the high prevalence of depression and anxiety in patients with CHF, there is a paucity of data on this topic as well as their impact on CHF outcomes in patients enrolled in the Balkans countries (and to a smaller extent from Germany) [11, 19, 23–26]. The aim of this study was to examine the impact of depression, anxiety and QoL on the occurrence of rehospitalization withinone year after discharge of patients diagnosed with CHF.

## Methods

MOLITOR (Impact of Therapy Optimization on the Level of Biomarkers in Patients with Acute and Decompensated Chronic Heart Failure) was a ten-center (eight in Serbia, one in Slovenia, and one in Germany) prospective study of 160 patients presenting to the emergency department with a primary diagnosis of CHF [18]. The study is registered under http://clinicaltrials.gov (NCT01501981), sponsored by Charité – Universitätsmedizin Berlin, and financed by an unrestricted grant by BRAHMS AG (Neuendorfstr. 25, 16,761 Hennigsdorf, Germany).

### Study design, population & instruments

Patients participating in the MOLITOR study with complete HADS and SF-36 data comprised the study group (n = 148, out of total 160 enrolled patients).The main inclusion criteria were [27]:

1. age ≥ 18 years.
2. HF with dyspnea at rest or minimal exertion (class III–IV according to New York Heart Association [NYHA])
3. pulmonary congestion.

Patients were excluded if they had an alternative diagnosis that could account for the HF symptoms, for example:

1. significant pulmonary disease (history of CO2 retention, or need for intubation for acute exacerbation, history of oral daily steroid dependency).
2. sepsis or active infection requiring intravenous (i.v.) antimicrobial treatment.
3. hypertrophic obstructive, restrictive, or constrictive cardiomyopathy.
4. significant arrhythmias (ventricular tachycardia, bradyarrhythmia with ventricular rate < 45 b.p.m., or atrial fibrillation/flutter with ventricular response of > 150 b.p.m.)
5. suspected acute myocardial infarction.
6. cardiogenic shock.
7. significant kidney disease with current or planned hemofiltration or dialysis.
8. or were younger than 18 years of age.

The full list of inclusion and exclusion criteria is listed elsewhere (NCT01501981 at the webpage of clinical trials.gov and ref [27]). In summary, patients hospitalized with symptomatic HF who did not meet any of the exclusion criteria were considered for enrollment. All patients in the study underwent study procedures such as: Echocardiography, blood collections for cardiac biomarkers and Six-minute walk test distance on protocol-predefined time points. At discharge from their primary hospitalization, patients also filled out the SF-36questionnaire and the Hospital Anxiety and Depression Scale (HADS). Any rehospitalization due to heart failure in the follow-up period of one year was recorded. The data was collected by the study team members and monitored independently. Written informed consent was obtained from all patients allowing the use of their data for future research purposes.

### Questionnaire Short Form Health Survey 36

Questionnaire Short Form Health Survey 36 (SF-36) is a generic multidimensional instrument for assessing patient health. This questionnaire consists of eight components that address General Health perception (GH), Physical Functioning (PF), Role Physical Functioning (RP), Vitality (VT), Physical Pain (BP), Emotional Role Functioning (RE), Social Functioning (SF) and Mental Health (MH) [21]. SF-36 produces eight scaled scores and the sum of the questions in each section is directly transformed into a scale of 0–100 assuming that each question has equal weight [22]. It is important to note that theQoL reflects the subjective perception of patients, so in patients with heart failure, it should be assessed appropriately to determine its impact on daily life [20]. The German [28–32] and Serbian versions of the SF-36 [33] were used to measure health-related QoL, both translations of questionnaires were derived from the original American SF-36 questionnaire, translated using a forward-backward method and are equal in their validity and relibility. The SF-36 consists of eight subscales that can be combined to a mental and a physical component score (MCS and PCS, respectively). MCS and PCS are standardized to US-population norms, with a mean of 50 and a standard deviation of 10. Higher scores reflect better QoL.

### Hospital anxiety and depression scale

HADS [34, 35] includes seven items for anxiety (HADS-Anxiety) and seven items for cognitive-affective features of depression (HADS-Depression). Anxiety and depression scores are obtained by summing up the scores of the seven items, yielding values between 0 and 21 (Higher scores indicate more psychological distress, while scores 0 to 7 were considered as Normal, 8 to 10 as Borderline, and 11 to 21 as Abnormal). Serbian and German translation of the scale were obtained from the Mapi Research Trust and are equal in their validity and reliability. This scale is to date widely used in clinical studies, which have demonstrated the validity, reliability, and usefulness of the HADS [34–36].

### Statistical analysis

Data are presented as mean ± standard deviation, or as counts and percentages. The unpaired Student’s t-test or Mann-Whitney test was used to compare continuous data, as appropriate. Chi-squared test or Fisher`s test was used to analyze categorical data. An exploratory logistic regression analysis was conducted to assess the significant associations between SF-36 scores further, and from these analyses, those variables with P < 0.10 were retained for the subsequent multivariable model (Backward Wald method). The Hosmer-Lemeshow test was performed to estimate calibration ability in the models. A complete case analysis was performed. A p-value was set at p < 0.05. All statistical analyses were performed using R software, version 3.4.3 (R Foundation for Statistical Computing, Vienna, Austria). The outcome between patients with high and low quality of life differed; some patients were rehospitalized over time or died due to cardiovascular and non-cardiovascular causes. Therefore, we used logistic regression to identify the quality of life components (independent variables) as predictors of potential rehospitalization (dependent variable). This allows for better explaining and predicting events, and identifying patients who will have low QoL after 1 year, already at an earlier point in time. As this study contains data from multiple centers, we have studied the factor ‘center’ with respect to its influence on the data.

## Results

### Socio-Demographic characteristics of the study population and occurrence of depression and anxiety

The study included 148 subjects, mean age 68.20 ± 10.12, 77.7% with NYHA III, 42.6% with *Diabetes mellitus* (DM type 1 and 2); 51.4% non-smokers, and 48.0% with 30–45% left ventricular ejection fraction (LVEF) (Table 1). The patients were enrolled at ten centers and three countries, however the majority of patients completing the follow-up period were from Serbian centers (Serbia 142, Slovenia 3, Germany 3) A total of 29 died during the 365-day follow-up period (19 of cardiac, 7 of noncardiac and 3 of unknown causes), of which 10 were rehospitalized. In total, 38 patients were rehospitalized in this period, with primary cause of rehospitalization being worsening of heart failure, as specified by the treating physician. None of the examined demographic and clinical parameters showed statistically significant (p < 0.05) differences in relation to rehospitalization occurrence (Table 1). We thus find no evidence for an association between demographics and clinical states and rehospitalization.

**Table 1:** Demographic and clinical characteristics of patients in relation to rehospitalization

Variable	Total		Rehospitalization		No rehospitalization		p^1^
**Age**	68.20 ± 10.12		68.36 ± 9.65		68.05 ± 10.23		0.410^2^
	** N of patients**	**Col%**	**N of patients**	**Row%**	**N of patients**	**Row%**	
**Gender**							
Male	104	70.3	29	27.9	75	72.1	0.459
Female	44	29.7	9	20.5	35	79.5	
**Marital status**							
divorced	2	1.4	0	0.0	2	100.0	0.162
married. living apart from spouse	4	2.7	3	75.0	1	25.0	
married. living together with spouse	101	68.2	26	25.7	75	74.3	
single	2	1.4	1	50.0	1	50.0	
widowed	39	26.4	8	20.5	31	79.5	
**Education** ^**3**^							
grade 0	10	6.8	3	30.0	7	70.0	0.783
grade 1	81	54.7	23	28.4	58	71.6	
grade 2	48	32.4	10	20.8	38	79.2	
grade 3	9	6.1	2	22.2	7	77.8	
**NYHA**							
III	115	77.7	28	24.3	87	75.7	0.642
IV	33	22.3	10	30.3	23	69.7	
**DM**	63	42.6	19	30.2	44	69.8	0.376
**COPD**	27	18.2	8	29.6	19	70.4	0.852
**Smoker**							0.360
Current	22	14.9	3	13.6	19	86.4	
ex-smoker (6 mon. clean)	48	32.4	15	31.3	33	68.8	
no (never smoked)	76	51.4	19	25.0	57	75.0	
Unknown	2	1.4	1	50.0	1	50.0	
**LVEF group**							0.355
< 30%	49	33.1	13	26.5	36	73.5	
30–45%	71	48	20	28.2	51	71.8	
45–55%	19	12.8	2	10.5	17	89.5	
≥ 55%	9	6.1	3	33.3	6	66.7	
**Deaths**	29	19.6	10	34.5	19	65.5	0.330

^1^ Chi-square test, ^2^ unpaired t-test, ^3^ Education was coded 0–3, with 0 = no education, 1 = primary education, 2 = Abitur [prerequisite to attend university], 3 = university degree

COPD: chronic obstructive pulmonary disease, NYHA: New York Heart Association; LVEF: left ventricular ejection fraction, DM: Diabetes mellitus

Data are presented as n (%), mean ± SD.

*P < 0.05

Furthermore, we examined whether demographic data affected the occurrence of depression and anxiety. We observed that depression was significantly higher in women compared to men (p = 0.027), as well as in those who were not married (p < 0.001) (Table 2.). Similarly, anxiety was significantly more frequent in patients with COPD (p = 0.016). Thus, our data suggest that anxiety is more common in patients with COPD and depression is elevated in women and unmarried patients.

**Table 2:** Depression and anxiety by categories

Variable	Category	Depression	p	Anxiety	p
Level of depression/anxiety	normal	18 (12.2%)		2 (1.35%)	
	borderline	90 (60.8%)		24 16.22%	
	abnormal	40 (27.03)		122 (82.43%)	
Gender	male	9.35 ± 1.91	0.027*	12.30 ± 1.78	0.776
	female	9.98 ± 1.89		12.34 ± 2.24	
Marital status	Yes	9.16 ± 1.69	< 0.001*	12.19 ± 1.80	0.208
	No	10.34 ± 2.14		12.57 ± 2.15	
Education	grade 0	9.80 ± 2.66	0.276	11.80 ± 2.25	0.329
	grade 1	9.62 ± 1.94		12.31 ± 1.96	
	grade 2	9.23 ± 1.68		12.25 ± 1.86	
	grade 3	10.11 ± 2.09		13.22 ± 1.48	
NYHA	III	9.62 ± 1.82	0.240	12.32 ± 1.95	0.824
	IV	9.24 ± 2.22		12.27 ± 1.84	
DM	no	9.76 ± 1.92	0.903	12.33 ± 1.92	0.214
	yes	9.36 ± 1.91		12.29 ± 1.94	
COPD	yes	9.56 ± 1.99	0.431	11.70 ± 1.98	0.016*
	no	9.59 ± 1.89		12.57 ± 1.76	
	unknown	8.58 ± 2.07		10.43 ± 2.88	
Smoker	current	9.04 ± 1.68	0.523	12.45 ± 1.65	0.892
	ex-smoker (> 6 months clean)	9.54 ± 1.86		12.21 ± 1.91	
	no (never smoked)	9.71 ± 2.02		12.32 ± 1.98	
LVEF group	< 30%	9.71 ± 1.91	0.642	12.12 ± 1.81	0.733
	30–45%	9.48 ± 1.96		12.44 ± 2.04	
	45–55%	9.00 ± 1.60		12.16 ± 1.77	
	≥ 55%	10.11 ± 1.92		12.67 ± 2.06	

COPD: chronic obstructive pulmonary disease, LVEF: left ventricular ejection fraction, NYHA: New York Heart Association, DM: Diabetes mellitus, Education was coded 0–3, with 0 = no education, 1 = primary education, 2 = Abitur [prerequisite to attend university], 3 = university degree

Data are presented as n, or score (%), mean ± SD.

### The relation of the levels of depression and anxiety to rehospitalization

**Fig. 1:**
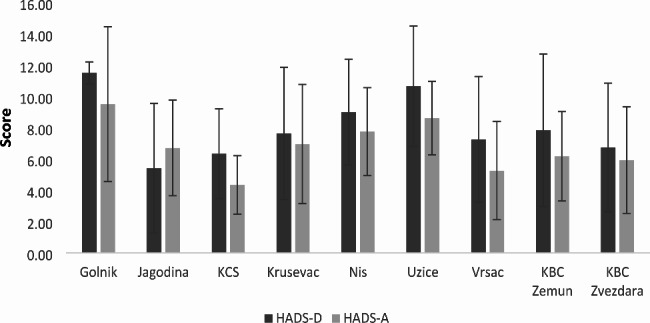
HADS-D (HADS-Depression) and HADS-A (HADS-Anxiety) among centers (mean value with ± standard deviation) KCS: Clinical Center Serbia; KBC Zvezdara: Clinical Hospital Center Zvezdara

The HADS measurements of anxiety (HADS-Anxiety) and depression (HADS-Depression) were significantly different among centers (p = 0.009, p = 0.001 respectively). The highest depressivness (mean of 11.5), as well as the highest anxiety (mean of 9.5), was in University Clinic Golnik (Golnik, Slovenia), while the lowest depression was in General Hospital Jagodina (Jagodina, Serbia) (mean of 5.4), and anxiety in Clinical Center Serbia (CCS, Serbia) (mean of 4.33) (Fig. 1). Due to the low number of patients who completed follow-up, the Charite Campus Virchow-Klinikum Center (Berlin, Germany) was left out of this analysis. However, due to the different numbers of patients in the centers, it is difficult to draw a relevant conclusion about these differences.

The HADS-Anxiety and HADS-Depression scores were not different in patients with and without rehospitalization (Table 3. HADS-Anxiety 12,40 ± 1,86 vs. 12,28 ± 1,95 and HADS-Depression 9,84 ± 1,85 vs. 9,43 ± 1,94 for rehospitalization vs. no rehospitalization respectively) indicating that the levels of anxiety and depression do not correlate with the risk of CHF worsening.

### The impact of quality of life on rehospitalization

We proceeded to examine the number of rehospitalized patients and the difference in their QoL at the time of discharge (Dis) (Table 3). Among eight scales of SF-36, Body pain, General health, Vitality and Social function showed significantly lower values in rehospitalized patients (p = 0.014, p = 0.002; p = 0.005 and p = 0.007, respectively). PCS and MCS were also significantly lower in patients who were rehospitalized (p = 0.008; and p = 0.007, respectively). We conclude that rehospitalization is more common in those with poorer quality of life, more precisely, reduced BP, GH, VT, SF, PCS andMCS.

**Table 3:** Characteristics of QoL in patients with CHF according to rehospitalization

	Rehospitalization	No rehospitalization	p
SF-36 Physical function (PF) (0-100)	38.42 ± 19.94	44.13 ± 22.76	0.272
SF-36 Role limitation due to physical problems / Role –Physical (RP) (0-100)	5.92 ± 19.65	9.77 ± 24.44	0.284
SF-36 Bodily pain (BP) (0-100)	48.03 ± 21.71	58.04 ± 22.3	0.014*
SF-36 General health (GH) (0-100)	34.97 ± 15.55	44.19 ± 17.11	0.002*
SF-36 Vitality (VT) (0-100)	28.42 ± 21.6	38.14 ± 20.11	0.005*
SF-36 Social function (SF) (0-100)	39.14 ± 19.1	49.77 ± 20.39	0.007*
SF-36 Role limitation due to emotional problems / Role Emotional (RE) (0-100)	19.3 ± 36.04	34.55 ± 43.99	0.066*
SF-36 Emotional well-being/ Mental health (MH) (0-100)	51.89 ± 16.23	53.96 ± 17.15	0.256
PCS - Physical component score	31.84 ± 14.11	39.03 ± 14.85	0.008*
MCS - Mental component score	34.67 ± 17.55	44.10 ± 20.09	0.007*
HADS-Anxiety	12,40 ± 1,86	12,28 ± 1,95	0,227
HADS-Depression	9,84 ± 1,85	9,43 ± 1,94	0,778

HADS: Hospital anxiety and depression scale; MCS: Mental component scale of the SF-36; PCS: Physical component scale of the SF-36.

### Prediction of the rehospitalizations during the 1-year follow-up by *QoL at discharge*

We calculated the odds ratios (OR) and their corresponding 95% confidence intervals (CI) of rehospitalization by use of logistic regression modelsbased on the initial values at the start of the prospective cohort (Tables 4 and 5). Using univariate logistic regression analysis, rehospitalization with one year was negatively associated with SF-36 Bodily Pain (OR 0.979, p = 0.020), SF-36 General Health (OR 0.966, p = 0.005), SF-36 Vitality (OR 0.976, p = 0.015), SF-36 Social Function (OR 0.974, p = 0.007). There was no difference in gender, age, Physical Function (PF), Role Limitation due to Physical Problems (RP), Role Limitation due to Emotional Problems (RE) and HADS in patients who were rehospitalized. In the multivariate model, GH (OR 0.966, p = 0.004) remained a significant risk factor for rehospitalization within a one-year follow-up (Table 4). Our results suggest that patients with impairedQoL are rehospitalized at increased rates.

**Table 4:** Risk factors for rehospitalization

	Univariate			Multivariate		
Risk factor	OR	95%CI	p	OR	95%CI	p
Gender	1.504	0.644–3.514	0.346	0.506	0.197–1.301	0.157
Age	1.016	0.978–1.056	0.408	1.015	0.973–1.059	0.495
SF-36 Physical function (PF)	0.988	0.971–1.005	0.172			
SF-36 Role limitation due to physical problems / Role –Physical (RP)	0.992	0.973–1.011	0.386			
SF-36 Bodily pain (BP)	0.979	0.962–0.997	0.020*	0.992	0.970–1.014	0.458
SF-36 General health (GH)	0.966	0.944–0.990	0.005*	0.966	0.944–0.990	0.005*
SF-36 Vitality (VT)	0.976	0.957–0.995	0.015*	0.996	0.969–1.025	0.805
SF-36 Social function (SF)	0.974	0.955–0.993	0.007*	0.987	0.961–1.014	0.334
SF-36 Role limitation due to emotional problems / Role Emotional (RE)	0.991	0.981-1.000	0.060			
SF-36 Emotional well-being/ Mental health (MH)	0.993	0.971–1.015	0.514			
HADS-Anxiety	1.064	0.971–1.166	0.182			
HADS-Depression	1.053	0.944–1.175	0.354			

Hosmer-Lemeshow p = 0.372, Backward Wald method, OR: Odds ratio HADS: Hospital anxiety and depression scale; MCS: Mental component scale of the SF-36; PCS: Physical component scale of the SF-36.

Furthermore, in the group with rehospitalization, there is a statistically significant association of MCS with HADS depression (r = -0.406, p = 0.011). In the group without rehospitalization, there is a statistically significant association of PCS with HADS depression (r = -0.347, p < 0.001). PCS was statistically significantly correlated with MCS in both groups (r = 0.792, p < 0.001) and (r = 0.635, p < 0.001, respectively) (Table 5). In the group without rehospitalization, there is a statistically significant association between age and HADS depression (r = 0.335, p < 0.001).

**Table 5:** Association of HADS-Depression, HADS-Anxiety and PCS and MCS in relation to the presence of rehospitalization

		Rehospitalization				No rehospitalization			
		HADS anxiety	PCS	MCS	Age	HADS anxiety	PCS	MCS	Age
HADS-Depression	r	-0,075	-0,291	-0,406*	0,286	0,046	-0,347**	-0,078	0,335**
	p	0,654	0,076	0,011	0,082	0,0636	< 0.001	0,416	< 0,001
	N	38	38	38	38	110	110	110	110
HADS-Anxiety	r		0,182	0,091	0,023		-0,048	0,169	-0,009
	p		0,271	0,585	0,892		0,616	0,077	0,927
	N		38	38	38		110	110	110
PCS	r			0,792**	-0,080			0,635**	-0,048
	p			< 0,001	0,635			< 0,001	0,616
	N			38	38			110	110

r – coefficient of correlation HADS: Hospital anxiety and depression scale; MCS: Mental component scale of the SF-36; PCS: Physical component scale of the SF-36.

### Prognostic value of multi-component questionnaire SF-36 for rehospitalization

**Fig. 2:**
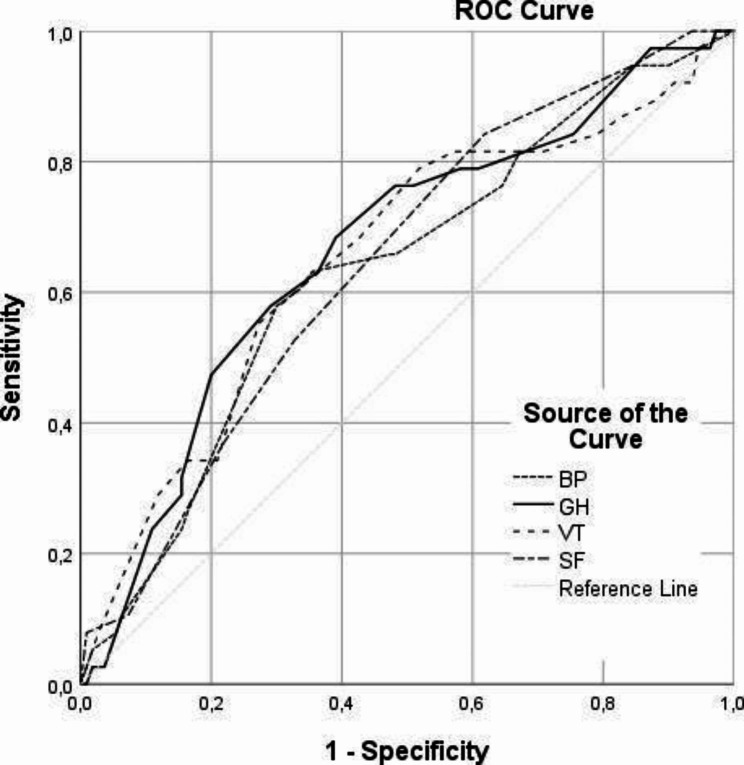
Receiver operating characteristic plot demonstrating the capacity of the four multi-component of Questionnaire SF-36 at hospital admission to predict one-year rehospitalization in 148 patients with acute heart failure; BP: Bodily pain; GH: General health; VT: Vitality; SF: Social function

We investigated the discriminative ability of SF-36 components for rehospitalization in one year using the receiver operating characteristic curves (Fig. 2). All four multi-component of Questionnaire SF-36 have significant and similar discriminative ability with AUC (Area Under the Curve) in range 0.632–0.665 (Table 6). A higher AUC indicates that General health has the best discriminative ability among all tested parameters (AUC = 0.665, P = 0.002) with corresponding cut-off 41.0, sensitivity 68.4% and specificity 60.9%. These results suggest that although none of the selected parameters has a strong predictive value, poor General Health is singled out as the most reliable prognostic parameter for rehospitalization.

**Table 6:** Prognostic value of multi-component of Questionnaire SF-36 for rehospitalization

Test Result Variable(s)	AUC	95% CI	SE	Cutoff	Sensitivity	Specificity	p
**BP**	0.632	0.53–0.735	0.052	51.50	63.2%	64.5%	0.015
**GH**	0.665	0.564–0.766	0.052	41.0	68.4%	60.9%	0.002*
**VT**	0.653	0.548–0.758	0.054	27.5	55.3%	72.7%	0.005
**SF**	0.644	0.547–0.741	0.05	43.75	52.6%	67.3%	0.008

AUC – area under ROC curve; SE – standard error; HADS: Hospital anxiety and depression scale; MCS: Mental component scale of the SF-36; PCS: Physical component scale of the SF-36.

## Discussion

In the current analyses, the MOLITOR trial data set was used to investigate the correlation among depression, anxiety, QoL and rehospitalization in patients with CHF. The key findings from this work are that levels of depression and anxiety did not correlate with rehospitalization in these patients, while QoL and especially component GH were strongest predictors of rehospitalization. Additionally, we identified that unmarried patients and female patients were more prone to have higher levels of depression, while cooccurrence of COPD was associated with higher levels of anxiety.

CHF is often associated with some of the symptoms of depression and anxiety [3, 5, 37]. Due to the similarities in physical and cognitive symptoms of depression and CHF (e.g., low energy, fatigue, sleep disturbance, weight loss or gain), the diagnosis of depression in patients with CHF may be difficult [11, 37]. While CHF diagnosis is supported by an array of findings (biomarkers, signs and symptoms, imaging), the diagnosis of depression is derived from clinical interview and questionnaires, which further complicates differentiation of a normal reaction to chronic illness from depression [10]. Some of the proposed mechanisms of influence of depression and anxiety on CHF are lack of motivation and adherence to healthy lifestyles (smoking, physical inactivity), dysregulation of inflammation ,as well as decreased compliance to pharmacological therapy. On the other hand, drugs used in the treatment of CHF can induce adverse effects (like fatigue, sexual dysfunction, gynecomastia, and body dysphoria) which can contribute to development of depression/anxiety ( [10]42). All these symptoms affect the quality of patients’ lives and reduce physical activity, which in a vicious circle leads to obesity, accelerated atherosclerosis, and coronary heart disease. These pathological conditions are the main risk factors for the development of CHF [4].

In this work, no association between demographic or clinical parameters and the occurrence of rehospitalizationwas observed (Table 1). In the analyzed group of patients, those without depression (normal) accounted for 18 (12.2%) patients, borderline (abnormal) 90 patients (60.8%) and depressed (abnormal) 40 (27.03%). This is in agreement with data from the literature, which reveal that these conditions affect 20–40% of patients with heart disease and occur 4–5 times more often than in the general population with 2–3 times higher mortality regardless of biological factors [3, 5, 38]. According to our results, depression was statistically significantly increased in women compared to men, as well as in unmarried patients, while patients with COPD showed significantly higher levels of anxiety (Table 2). Given that the largest number of respondents have been from Serbia, it is important to note that there is evidence that stress-related disorders are significantly increased in this population due to various stressors, which can further affect the occurrence of depression and anxiety [39–41]. Results from Germany were obtained from a small number of patients, so an adequate conclusion in terms of differences between countries cannot be drawn. In general, approximately 17–37% of patients who were hospitalized with HF have major depressive disorder while 16–22% had minor depression [3]. Several other studies report different ranges of CHF patients with depression spanning from 13 to 78% [24, 42, 43]. The differences in the depression prevalence in patients withCHF might be a consequence of different screening test or criteria for a diagnosis, as well as the severity of the clinical picture itself, which can mask the symptoms of depression. Living conditions, the organization of health care and the standard of living in different countries can have an additional impact. This emphasizes the importance of the universally accepted measurement instrument’s application that will allow comparisons between populations [44]. Similar results were obtained for anxiety. There were only 2 (1.35%) patients without anxiety, borderline abnormal 24 (16.22%) and those with anxiety (abnormal) 122 (82.43%). In our study, after 365 days, 38 patients (25.67%) were rehospitalized, while 29 (19.5%) died. In the meta-analysis of 8 studies, depression was shown to be a worsening prognostic factor for mortality, while some studies suggest that it might be an independent risk factor for poor prognosis in cardiac patients with higher mortality and rehospitalization [19].

Our results, however, do not support this notion, and depression and anxiety levels were not associated with the increased rehospitalization, in our study population and within the follow-up period. In contrast, a twenty-year follow-up study, by Freedland and coworkers demonstrated that the severity of depression is linked to mortality even years after the first hospitalization [45]. Liguori et al. showed that depression was a worsening factor only in CHF patients with left ventricular ejection fraction ≤ 35% [26] while Celano et al. [8] revealed that there is no notable association between mortality and anxiety in relation to medical and demographic variables which is consistent with the results of our study. These discrepancies in the reported results could be contributed to differences in the study population, inclusion and exclusion criteria, sample sizes or the duration of the follow-up period.

QoL depends on many factors such as socioeconomic status, societal support, associated diseases, the severity of the clinical picture and it is reduced in all parameters of the SF-36 scale [21]. QoL is seen in a broader sense, including all factors that directly or indirectly relate to health status. It reflects a person’s mental and physical well-being in his or her daily life. Since the functional status of CHF patients tends to affect the domains of QoL (physical, psychological, social, emotional, sexual and mental well-being), it is important to assess the patient’s condition as early as possible for timely interventions [18]. The parameters that define the quality of life, such as Bodily pain (BP), General health (GH), Vitality (VT) and social function (SF), are significantly associated with rehospitalization in our patients (Table 3). Physical and mental health were also worse in rehospitalized patients compared to those who were not rehospitalized. Gottlieb et al. studied the prevalence of depression in an outpatient heart failure population and its relationship to QoL. A total of 48% of patients were diagnosed with depression and their scores were notably worse than those in non-depressed patients which included all components of both questionnaires that measure QoL. In this study, depression was observed more commonly among younger than older patients [24]. Some of the symptoms characteristic for both depression and CHF, such as lack of energy, sleep disturbance, fatigue, weight loss, decreased concentration, and memory impairment are present in both groups of patients and significantly affect the QoL [26]. In the univariate regression analysis, BP, GH, VT and SF were segregated as statistically significant risk factors for rehospitalization, while in the multivariate model, only GH remained a significant risk factor. A study from Edinburgh revealed that patients with more severe symptoms, poor socio-economic status and low support by society have a poorer QoL, which also increases their chances of rehospitalization [46]. Poor QoL is predominantly experienced by younger men with poor socio-economic status, the upper NYHA class, with a mortality rate of 37% during three years of follow-up [47]. In general, the quality of life is significantly worse in these patients compared to the healthy population, and it is necessary to introduce routine screening of QoL for all patients with heart disease [48]. Patients with NYHA class III have a worsening QoL similar to those with major depression [21]. In Gottlieb et al. work, it was shown that all patients with heart failure, who were also diagnosed with depression, possess significant impairment of all physical and mental health parameters measured with SF-36 [24]. A key factor in providing high quality care to CHF patients is the continuous monitoring and appropriate providing of the necessary information that accompanies the disease phase, as in the early stages of the disease, which can improve both the physical and emotional status of these patients [49].

In our sample of patients, the overall physical and mental health were significantly impaired in those with rehospitalization compared to those who were not rehospitalized, as well as BP, GH, V, SF. The group of patients, rehospitalized during the one year long period, revealed a statistically significant correlation of MCS and depression, while in the group of patients that were not rehospitalized, the association between age and depression was established, where older patients showed a higher predisposition to depression. PCS was statistically associated with MCS in both groups of patients (Table 5). According to our study, General Health was the strongest predictor of rehospitalization (AUC = 0.665, P = 0.002), followed by VT (AUC = 0.653, P = 0.005) (Fig. 1). Initial QoL may be a predictor of adverse clinical outcomes such as short-term mortality, risk of early rehospitalization and length of hospitalization [38]. Mbakvem and coworkers found a negative correlation between quality of life and the number of rehospitalizations [50]. Adeba**y**o et al. indicate that the assessment of life quality provides additional predictive values ​​with respect to CHF-related mortality and hospitalizations, superior to the predictive power of variables such as EF, age, treatment, and NYHA class [18]. A study by Iqbal and colleagues over a 3-year follow-up period of time found that poor socioeconomic status and lack of social support result in poor quality of life in CHF patients, which leads to an increased risk of hospital admission and mortality that was 37% in this group [46]. Taken together, our results suggest that timely QoL assessment, should be introduced to screening of patients with CHF to recognize those with high risk for adverse events.

## Limitations

The major limitations of this study are the relatively small sample size and the moderate follow-up period. Furthermore, the majority of patients were recruited at sites in Serbia, while other countries contributed much less to the enrollment, making it harder to generalize the conclusions. Finally, the SF-36 Questionnaire even though being the standard in QoL measurement is not specific to the CHF patient population. It is possible that using disease-specific QoL measurement would lead to better evaluation of its impact on rehospitalization, but it would also make comparison to published studies more difficult. The majority of patients recruited in this study are male (104 vs. 44 of female patients) and married (101 out of 148), which is typical for cardiovascular clinical trials [51], however more balanced sample could benefit the quality of the results. Future studies with higher statistical power should be encouraged in order to further characterize components of depression and anxiety that could influence CHF patients’ outcomes.

## Conclusion

Screening for depression and anxiety in CHF patients and attention to persistent symptoms of depression and anxiety are important to identify patients that may need enhanced clinical treatment and support, especially in female and unmarried patients. Timely QoL assessment might complement clinical prognostic markers for the recognition of patients with CHF with high risk for adverse events that include depression and anxiety. Furthermore, successful treatment strategy should be guided by the QoL assessment and present a multidisciplinary approach with the involvement of psychiatrists, social workers, family, and society.

## Data Availability

The datasets used and/or analyzed in this study are available from the corresponding author on reasonable request.

## Abbreviations

AUC: Area under the curve
BP: Bodily pain
CHF: Chronic heart failure
CI: Confidence interval
COPD: chronic obstructive pulmonary disease
DM: Diabetes mellitus
GH: General health
HADS: The Hospital Anxiety and Depression Scale
HRQoL: Health related Quality of Life
LVEF: Left ventricular ejection fraction
MCS: Mental component score
MH: Mental health
MOLITOR: Impact of Therapy Optimization on the Level of Biomarkers in Patients with Acute and Decompensated Chronic Heart Failure
NYHA: New York Heart Association
OR: Odds ratio
PCS: Physical component score
PF: Physical functioning
QoL: Quality of Life
RE: Emotional role functioning
RP: Role physical functioning
SE: Standard error
SF: Social function
SF-36: Questionnaire Short Form Health Survey 36
VT: Vitality
